# Differential Proteomics Analysis of *Bacillus amyloliquefaciens* and Its Genome-Shuffled Mutant for Improving Surfactin Production

**DOI:** 10.3390/ijms151119847

**Published:** 2014-10-31

**Authors:** Junfeng Zhao, Lin Cao, Chong Zhang, Lei Zhong, Jing Lu, Zhaoxin Lu

**Affiliations:** 1 College of Food Science and Technology, Nanjing Agricultural University, Nanjing 210095, Jiangsu, China; E-Mails: zjf780526@sina.com (J.Z.); caolin@njau.edu.cn (L.C.); zhangchong@njau.edu.cn (C.Z.); 2011108029@njau.edu.cn (L.Z.); 2012108039@njau.edu.cn (J.L.); 2 College of Food Science and Engineering, Henan University of Science and Technology, Tianjing Road, Luoyang 471003, China

**Keywords:** *Bacillus amyloliquefaciens*, genome shuffling, surfactin, proteomics, MALDI-TOF/MS

## Abstract

Genome shuffling technology was used as a novel whole-genome engineering approach to rapidly improve the antimicrobial lipopeptide yield of *Bacillus amyloliquefaciens*. Comparative proteomic analysis of the parental ES-2-4 and genome-shuffled FMB38 strains was conducted to examine the differentially expressed proteins. The proteome was separated by 2-DE (two dimensional electrophoresis) and analyzed by MS (mass spectrum). In the shuffled strain FMB38, 51 differentially expressed protein spots with higher than two-fold spot density were detected by gel image comparison. Forty-six protein spots were detectable by silver staining and further MS analysis. The results demonstrated that among the 46 protein spots expressed particularly induced in the genome-shuffled mutant, 15 were related to metabolism, five to DNA replication, recombination and repair, six to translation and post-translational modifications, one to cell secretion and signal transduction mechanisms, three to surfactin synthesis, two to energy production and conversion, and 14 to others. All these indicated that the metabolic capability of the mutant was improved by the genome shuffling. The study will enable future detailed investigation of gene expression and function linked with surfactin synthesis. The results of proteome analysis may provide information for metabolic engineering of *Bacillus amyloliquefaciens* for overproduction of surfactin.

## 1. Introduction

*Bacillus* strains produce many types of bioactive lipopeptides that are synthesized non-ribosomally by a large multifunctional enzyme complex. Of these, the lipopeptide surfactin is well characterized at the genetic level. Surfactin is biosynthesized by three non-ribosomal peptide synthetases, SrfA–C, and by the thioesterase/acyltransferase enzyme SrfD, which initiates this process. Surfactin is a powerful biosurfactant that is known to decrease the surface tension of water [1]. It exerts a detergent-like action on biological membranes, and is distinguished by its emulsifying, foaming, antiviral and anti-mycoplasma activities. Surfactin has many potential applications in plant disease biocontrol [2] and biomedicine [3]. Moreover, lipopeptides are widely used in the food [4] and cosmetics industries [5], and for enhanced oil recovery [6] and bioremediation of oil-contaminated sites [7]. However, the production of antimicrobial peptides in *Bacillus* is generally less than 1.0 g/L, and even as low as 0.1 g/L for some peptides. Therefore, it is particularly important to improve antimicrobial peptide production in industrially important *Bacillus* strains. There have been many attempts to increase lipopeptide production, but almost all have focused on the optimization of fermentation [8], isolation and purification [9], or on the regulation of lipopeptide synthesis using genetic engineering methods [10,11]. Although global techniques have been successfully applied to strain improvement, engineering more complex phenotypes requires a more combinatorial approach.

The synthesis of antimicrobial substances in *Bacillus* species is closely related to the formation of competent cells and sporulation, with these three pathways sharing the same metabolic network [12]. Surfactin biosynthesis and regulation of competent cell formation are also closely linked. Interestingly, the *comS* gene, which is involved in competent cell formation, is located in the surfactin synthase gene *srfA* operon. The expression of *comS*, *srfA* and several quorum sensing genes is regulated by a complex network, which includes the extracellular ComX protein and the two-component adjustment system ComPA [12]. Phosphorylated ComA (ComA–P) was shown to promote *srfA* operon expression by binding to the promoter region. CodY and AbrB suppressed *srfA* operon expression by binding to the *srfA* promoter [13]. The signaling protein RapC could inhibit *srfA* expression by dephosphorylating ComA–P. Additionally, YerP could increase the tolerance for surfactin in *Bacillus subtilis* [14].

Genome shuffling involves generation of mutant strains that have an improved phenotype, followed by multiple rounds of protoplast fusion to allow recombination between genomes. A strain with a high yield of a desired product can rapidly be obtained by genome shuffling without knowledge of the metabolic regulatory mechanism. We previously described the generation of a high-yield recombinant *Bacillus amyloliquefaciens* F2-38 (FMB38) strain that exhibited 3.5- and 10.3-fold increases in surfactin production in a shake flask and fermenter, respectively, following two rounds of genome shuffling. Comparative analysis of synthetase gene expression was conducted between the parental and shuffled strains using FQ (fluorescent quantitation) RT-PCR. Δ*C*_t_ (threshold cycle) relative quantitation analysis revealed that surfactin synthetase gene (*srf*A) expression at the transcriptional level in the F2-38 strain was 15.7-fold greater than in the parental strain ES-2-4 [15]. However, these results only indirectly identified differences at the transcriptional level in the lipid peptide synthetase gene. Because proteins carry out molecular functions and are responsible for almost all the biochemical activitiesof the cell, a real understanding of biological systems requires the direct study of proteins. The rapid development of proteomics technology based on 2-DE, identification by MS, and bioinformatics provides a good platform for large-scale proteomic studies. In this study, we explored the molecular mechanism of high-yield surfactin using comparative proteomics methods to identify the differentially expressed proteins between the parental and mutant strains.

## 2. Results

### 2.1. Identification of Differentially Expressed Proteins

**Figure 1:**
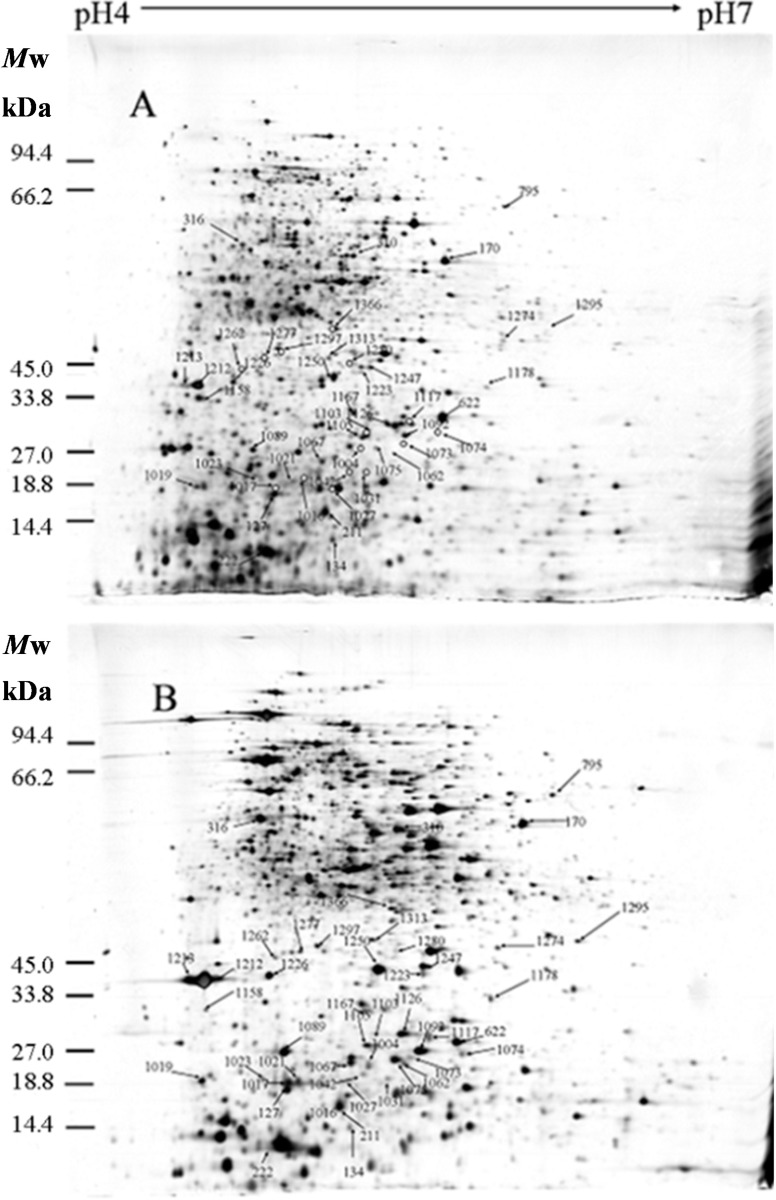
2-DE maps of the differentially regulated cellular proteins (>2-fold change in expression) of *Bacillus amyloliquefaciens* FMB38. (**A**) ES-2-4; and (**B**) FMB38.

We analyzed the 2-DE profiles of soluble proteins from parental (ES-2-4) and mutant (FMB38) strains of *B. amyloliquefaciens* and found 51 protein spots that differed between the strains by more than two-fold (Figure 1). These 51 protein spots were identified by MS analysis and their complete peptide fingerprints were obtained. Searching of the NCBI nr database with Mascot revealed that protein spots 795 and 816, 1004 and 1056, 1062 and 1065, 1117 and 1120, and 1213 and 1220 were the same proteins, meaning that a total of 46 proteins were successfully identified. In *B. amyloliquefaciens* FMB38, 29 proteins had increased expression, two proteins had decreased expression, and 15 proteins appeared only in this strain (Table 1).

### 2.2. Isoelectric Point and Molecular Weight Analysis of Theoretically and Experimentally Identified Proteins

Isoelectric points and molecular weights of the identified proteins were determined using analysis software (ImageMaster 2D). Samples were compared with the migration distances of the molecular weight marker, and the pH of the IPG (immobilized pH gradient) strips was used to determine the isoelectric point of the protein spots in the silver stained gel. These values were compared with proteins in the database to assist with identification. Overall, the gel estimated molecular weight and isoelectric point of each protein closely matched the theoretical values, but there were some proteins for which the estimated molecular weight (spots 1031, 1074, 1313, and 1366) or isoelectric point (spots 1023, 1031, 1073, 1074, 1075, and 1262) differed greatly from the theoretical value (Table 1).

**Table 1:** Identification of differentially regulated cellular proteins (>2-fold change in expression) of *Bacillus amyloliquefaciens* FMB38.

Spot No. ^a^	Protein Name ^b^	Accession No. ^c^	Locus ^d^	Gene ^e^	Theor. ^f^ Mr/pI	Exper. ^g^ Mr/pI	Protein Score ^h^	Sequence Coverage (%) ^i^	Fold Change ^j^ (*p* < 0.05)
127	Alkyl hydroperoxide reductase small subunit	gi|308175696	YP_003922401	*ahpC*	20,669/4.51	21,013/4.65	104	44	+15.3
134	Conserved hypothetical protein	gi|315173048	EFU17065	*–*	14,048/5.12	13,978/5.10	158	55	−18.4
170	DNA primase	gi|228983124	ZP_04143383	*–*	55,979/5.93	56,328/5.79	115	48	−19.8
211	Hypothetical protein RBAM_036960	gi|154688095	YP_001423256	*ahpC*	20,683/4.51	17,985/4.62	126	50	+34.7
222	Hypothetical protein KSO_14324	gi|363725374	EHM05512	*–*	6927/4.56	6843/4.49	120	70	+2.7
310	Hypothetical protein RBAM_029780	gi|154687379	YP_001422540	*yurX*	48,265/5.30	48,965/5.33	109	32	+29.1
316	GroEL gene product	gi|311067075	YP_003971998	*groEL*	57,385/4.75	56,789/4.78	190	43	+6. 6
622	Response regulator DegU	gi|157693950	YP_003974978	*degU*	25,893/5.65	27,124/5.67	249	92	−6.1
795, 816	Vegetative catalase 1	gi|89097371	ZP_01170260	*–*	54,421/6.11	55,135/5.89	192	40	+7.5, +4.7
1004, 1056	Hypothetical protein RBAM_023340	gi|154686764	YP_001421925	*sodA*	22,365/5.21	23,214/5.43	316	80	in FMB38
1016	*S*-ribosylhomocysteinase	gi|154687196	YP_001422357	*luxS*	17,913/5.27	18,324/5.09	98	66	in FMB38
1017	Thiol peroxidase	gi|154687070	YP_001422231	*tpx*	18,262/4.99	17,321/4.89	180	89	in FMB38
1019	Hypothetical protein RBAM_026720	gi|154687100	YP_001422261	*yraA*	18,672/4.94	17,652/4.63	110	72	+26.2
1021	Hypothetical protein RBAM_008040	gi|154685258	YP_001420419	*yfkM*	18,877/4.83	18,896/4.85	163	85	+17.9
1023	Hypothetical protein RBAM_028130	gi|154687215	YP_001422376	*yuaE*	19,112/5.46	19,431/4.94	112	56	+4.7
1027	ATP-dependent Clp protease proteolytic subunit	gi|154687585	YP_001422746	*clpP*	21,874/4.96	19,543/5.12	154	60	in FMB38
1031	DNA-directed DNA polymerase III α subunit	gi|325684283	EGD26456	*dnaE*	128,931/8.83	19,678/5.34	81	18	in FMB38
1042	Methionine aminopeptidase, type I	gi|229010975	ZP_04168170	*–*	27,381/4.89		190	49	in FMB38
1062, 1065	Transaldolase	gi|154687826	YP_001422987	*tal*	23,055/5.23	23,336/5.31	86	40	+17.9, +6.5
1067	Hypothetical protein RBAM_036480	gi|154688047	YP_001423208	*deoC*	23,111/4.90	23,352/5.08	94	40	+24.0
1073	Hypothetical protein KSO_05864	gi|363723690	EHM03828	*–*	23,280/4.62	242,110/5.41	112	56	in FMB38
1074	2-Aminoethylphosphonate—Pyruvate transaminase	gi|229166259	ZP_04294018	*–*	41,797/5.29	27,312/5.61	220	50	in FMB38
1075	Recombinase protein	gi|339764913	AEK01094	*recA*	24,141/6.39	24,234/5.31	139	74	+8.3
1089	ComA	gi|154687277	YP_001422438	*comA*	24,371/5.19	24,351/4.91	280	51	+18.7
1092	Transcriptional repressor CodY	gi|154686033	YP_001421194	*codY*	29,038/4.90	28,213/5.4	110	47	+7.7
1103	Pyrroline-5-carboxylate reductase	gi|228921645	ZP_04084963	*–*	29,356/5.37	28,531/5.31	77	45	in FMB38
1105	NAD synthetase NadE	gi|302671492	YP_003831452	*nadE*	29,690/5.00	28,921/5.12	87	37	+6.7
1117, 1120	Fructose-bisphosphate aldolase	gi|154687827	YP_001422988	*fbaA*	30,537/5.26	29,314/5.41	103	34	in FMB38
1126	Hypothetical protein RBAM_036720	gi|154688071	YP_001423232	*iolG*	38,449/5.14	37,111/5.41	225	38	+18.6
1158	Putative glycerol-3-phosphate acyltransferase PlsX	gi|326941622	AEA17518	*plsX*	35,512/6.27	35,212/5.81	79	51	+5.1
1167	Glyceraldehyde-3-phosphate dehydrogenase (phosphorylating)	gi|157693809	YP_001488271	*gapA*	35,822/5.03	35,344/5.13	92	29	+4.0
1178	Unnamed protein product	gi|311069921	YP_003974844	*–*	35,900/5.10	35,212/5.82	93	33	+164.7
1212	Hypothetical protein RBAM_006650	gi|154685120	YP_001420281	*ydjL*	37,552/5.09	35,242/4.57	91	29	+3.5, +4.9
1213, 1220	YdjL	gi|363726006	EHM06144	*–*	37,580/5.04	36,899/4.59	86	31	+14.6
1223	Multifunctional SOS repair factor	gi|308173657	YP_003920362	*recA*	37,938/5.05	37,432/5.21	148	57	+53.3
1226	30S ribosomal protein S1	gi|328552793	AEB23285	*rpsA*	41,960/4.82	41,556/4.79	81	22	+16.8
1247	Elongation factor Tu	gi|154684631	YP_001419792	*tufA*	43,500/4.84	43,211/5.23	96	37	+5.5
1250	Hypothetical protein Dtox_1245	gi|258514528	YP_003190750	*–*	44,823/5.15	44,112/5.16	81	37	+12.1
1262	Site-specific recombinase XerD	gi|295101383	CBK98928	*–*	46,358/9.08	45,212/4.81	77	43	in FMB38
1274	Plasmid recombination protein	gi|10956056	NP_042279	*pre*	49,739/5.24	48,677/5.84	136	48	+11.5
1277	Hypothetical protein RBAM_007590	gi|154685214	YP_001420375	*yfmT*	53,319/5.26	52,695/4.82	113	29	in FMB38
1280	F0F1 ATP synthase subunit α	gi|154687798	YP_001422959	*atpA*	54,804/5.34		113	26	in FMB38
1295	Galactose-1-phosphate uridylyltransferase	gi|363725414	EHM05552	*–*	56,804/6.27	56,123/6.16	301	54	+9.2
1297	Phosphopyruvate hydratase	gi|154687527	YP_001422688	*eno*	46,645/4.68	46,131/4.85	300	61	in FMB38
1313	Dak2 domain fusion protein ylov	gi|312135039	YP_004002377	*–*	60,930/4.99	43,123/5.10	77	23	+7.0
1366	M6 family metalloprotease	gi|172058939	YP_001815399	*–*	86,115/5.33	48,531/5.21	91	21	in FMB38

^a^ Spot numbers assigned by the software refer to the proteins labeled in Figure 1; ^b^ Protein name in the National Center for Biotechnology Information (NCBI) database for *Bacillus amyloliquefaciens*; ^c^ Accession number in the NCBI database for *Bacillus amyloliquefaciens*; ^d^ The specific location of a gene or DNA sequence on of the *Bacillus amyloliquefaciens* chromosome; ^e^ Gene designation in the NCBI database for *Bacillus amyloliquefaciens*; ^f^ Theoretical molecular mass (Mr) and isoelectric point (pI) were obtained from the protein database in the NCBI database for *Bacillus amyloliquefaciens*; ^g^ Experimental molecular mass (Mr) and isoelectric point (pI) were obtained from the 2-DE gels; ^h^ MASCOT protein score from MS; ^i^ Percentage of amino acids in reference proteins covered by matching peptides from MS; and ^j^ Fold change: positive values represent over-expressed proteins, negative values represent under-expressed proteins, “in FMB38” indicates that the protein appeared only in high-yield strain FMB38.

### 2.3. Cellular Localization Analysis of Experimentally Identified Proteins

**Figure 2:**
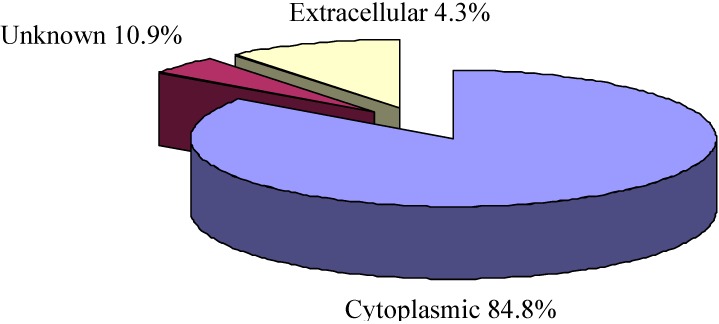
Cellular localization of the differentially expressed proteins identified in *Bacillus amyloliquefaciens* FMB38 predicted by the PSORTb database.

PSORTb version 3.0.1 (http: //www.psort.org/psortb/index.html) was used to predict the cellular localization of the 46 identified proteins (Table 2). Thirty-nine proteins localized to the cytoplasm, two proteins were extracellular, and five proteins had an unknown cellular location (Table 2). The separated proteins are mainly the proteins in the cytoplasm, accounting for 84.8% of the total proteins (Figure 2).

### 2.4. Classification and Functional Analysis of Differential Proteins

Experimentally identified proteins were functionally characterized using clusters of orthologous groups (COG) analysis (Table 2). The isolated proteins were mainly divided into the following categories: Energy production and conversion (C); Cell division and chromosome distribution (D); Amino acid transport and metabolism (E); Nucleic acid transport and metabolism (F); Carbon transport and metabolism (G); Coenzyme metabolism (H); Lipid metabolism (I); Translation, ribosomal structure, and biosynthesis (J); DNA replication, recombination, and repair (L); Cell motility and secretion (N); Post-translational modification, protein folding, chaperone proteins (O); Inorganic ion transport and metabolism (P); Secondary metabolites biosynthesis, transport, and catabolism (Q); General function prediction (R); Signal transduction mechanisms (T); and not included in the COG classification (−). To determine the mechanism of increased antimicrobial peptide yield from *B. amyloliquefaciens* FMB38, biological process and molecular function data were obtained from the UniProKB (http://www.uniprot.org) (accessed on 1 October 2014) database [16].

**Table 2:** Cellular localization and function of differentially regulated cellular proteins (>2-fold change in expression) of *Bacillus amyloliquefaciens* FMB38.

Spot No. ^a^	Protein Name ^b^	COG ^c^	Cellular Localization ^d^	Biological Process ^e^	Molecular Functional Annotation ^f^
**Energy Production and Conversion**					
1277	Hypothetical protein RBAM_007590	C	Cytoplasmic	Unknown	Oxidoreductase activity, acting on the aldehyde or oxo group of donors, NAD or NADP as acceptor
1280	F0F1 ATP synthase subunit α	C	Cytoplasmic	ATP hydrolysis coupled proton transport, plasma membrane ATP synthesis Coupled proton transport	Hydrogen ion transporting ATP synthase activity, rotational mechanism; hydrolase activity
**Cell Division and Chromosome Partitioning**					
1274	Plasmid recombination protein	D	Cytoplasmic	DNA recombination	DNA binding
**Amino Acid Transport and Metabolism**					
1074	2-Aminoethylphosphonate—Pyruvate transaminase	E	Cytoplasmic	Organic phosphonate catabolic process	2-Aminoethylphosphonate-pyruvate transaminase activity, pyridoxal phosphate binding
1103	Pyrroline-5-carboxylate reductase	E	Cytoplasmic	Proline biosynthetic process	Nucleotide binding, oxidoreductase activity, acting on the CH–OH group of donors, NAD or NADP as acceptor, pyrroline-5-carboxylate reductase activity
1212	Hypothetical protein RBAM_006650	ER	Cytoplasmic	Unknown	Nucleotide binding, oxidoreductase activity, zinc ion binding
1213, 1220	YdjL	ER	Cytoplasmic	Unknown	Nucleotide binding, oxidoreductase activity, zinc ion binding
**Nucleotide Transport and Metabolism**					
1067	Hypothetical protein RBAM_036480	F	Cytoplasmic	Deoxyribonucleotide catabolic process	Deoxyribose-phosphate aldolase activity
**Carbohydrate Transport and Metabolism**					
1062, 1065	Transaldolase	G	Cytoplasmic	Pentose-phosphate shunt	Sedoheptulose-7-phosphate: d-glyceraldehyde-3-phosphate glyceronetransferase activity
1117, 1120	Fructose-bisphosphate aldolase	G	Cytoplasmic	Fructose 1,6-bisphosphate metabolic process, glycolysis, sporulation resulting in formation of a cellular spore	Fructose-bisphosphate aldolase activity, zinc ion binding
1167	Glyceraldehyde-3-phosphate dehydrogenase (phosphorylating)	G	Cytoplasmic	Glycolysis	NAD binding; NADP binding, glyceraldehyde-3-phosphate dehydrogenase (NAD^+^) (phosphorylating) activity
1178	Unnamed protein product	G	Cytoplasmic	Unknown	Glyceraldehyde-3-phosphate dehydrogenase/erythrose-4-phosphate dehydrogenase
1297	Phosphopyruvate hydratase	G	Cytoplasmic	Glycolysis	Magnesium ion binding, phosphopyruvate hydratase activity
**Coenzyme Metabolism**					
1105	NAD synthetase NadE	H	Unknown	NAD biosynthetic process, response to stress, sporulation resulting in formation of a cellular spore	ATP binding, NAD^+^ synthase (glutamine-hydrolyzing) activity, NAD^+^ synthase activity
**Lipid Metabolism**					
1158	Putative glycerol-3-phosphate acyltransferase PlsX	I	Unknown	Phospholipid biosynthetic process	Transferase activity, transferring acyl groups other than amino-acyl groups
**Translation, Ribosomal Structure, and Biogenesis**					
1042	Methionine aminopeptidase, type I	J	Cytoplasmic	Protein initiator methionine removal, proteolysis	Ferrous iron binding, metalloaminopeptidase activity
1226	30S ribosomal protein S1	J	Cytoplasmic	Translation	RNA binding, structural constituent of ribosome
1247	Elongation factor Tu	JE	Cytoplasmic	Response to antibiotic	GTP binding, GTPase activity, protein binding, translation elongation factor activity
**DNA Replication**					
170	DNA primase	L	Cytoplasmic	DNA replication, synthesis of RNA primer	ATP binding; DNA binding, DNA helicase activity, DNA primase activity, zinc ion binding
1031	DNA-directed DNA polymerase III α subunit	L	Cytoplasmic	Transcription	Nucleotidyltransferase, transferase
1075	Recombinase protein	L	Cytoplasmic	DNA recombination, DNA repair, SOS response	ATP binding, DNA-dependent ATPase activity, single-stranded DNA binding
1223	Multifunctional SOS repair factor	L	Cytoplasmic	DNA recombination, DNA repair, SOS response	ATP binding, DNA-dependent ATPase activity, damaged DNA binding, single-stranded DNA binding
1262	Site-specific recombinase XerD	L	Cytoplasmic	DNA integration, DNA recombination	DNA binding
**Cell Motility and Secretion**					
1027	ATP-dependent Clp protease proteolytic subunit	NO	Cytoplasmic	Protein metabolic process	ATP binding, ATP-dependent peptidase activity, protein binding
**Posttranslational Modification**					
127	Alkyl hydroperoxide reductase small subunit	O	Cytoplasmic	Unknown	Cytochrome-c peroxidase activity, peroxiredoxin activity
211	Hypothetical protein RBAM_036960	O	Cytoplasmic	Unknown	Peroxidase activity; peroxiredoxin activity
316	GroEL gene product	O	Cytoplasmic	Cellular protein metabolic process	ATP binding
**Inorganic Ion Transport and Metabolism**					
795, 816	Vegetative catalase 1	P	Cytoplasmic	Response to oxidative stress	Catalase activity, heme binding
1004, 1056	Hypothetical protein RBAM_023340	P	Extracellular	Superoxide metabolic process	Metal ion binding, superoxide dismutase activity
**Secondary Metabolite Biosynthesis, Transport, and Catabolism**					
1073	Hypothetical protein KSO_05864	Q	Cytoplasmic	Peptidyl-pyrromethane cofactor linkage, porphyrin-containing compound biosynthetic process	Hydroxymethylbilane synthase activity
**General Function Prediction**					
310	Hypothetical protein RBAM_029780	R	Unknown	Iron-sulfur cluster assembly	Unknown
1019	Hypothetical protein RBAM_026720	R	Cytoplasmic	Unknown	Hydrolase activity, acting on glycosyl bonds
1021	Hypothetical protein RBAM_008040	R	Cytoplasmic	Unknown	Hydrolase activity, acting on glycosyl bonds
1126	Hypothetical protein RBAM_036720	R	Cytoplasmic	Inositol Catabolic Process	Inositol 2-dehydrogenase activity, nucleotide binding
1313	Dak2 domain fusion protein ylov	R	Cytoplasmic	Glycerol metabolic process	Glycerone kinase activity
**Signal Transduction Mechanisms**					
622	Response regulator DegU	TK	Cytoplasmic	Transcription, DNA-dependent	Sequence-specific DNA binding, sequence-specific DNA binding transcription factor activity, two-component response regulator activity
1016	*S*-ribosylhomocysteinase	T	Cytoplasmic	Quorum Sensing	iron ion binding, lyase activity
1089	ComA	TK	Cytoplasmic	Transcription, DNA-dependent	Sequence-specific DNA binding, sequence-specific DNA binding transcription factor activity, two-component response regulator activity
**Others**					
134	Conserved hypothetical protein	–	Unknown	Growth of symbiont in host, protein omooligomerization	ATP binding, ATPase activity, protein binding
222	Hypothetical protein KSO_14324	–	Unknown	Unknown	Electron carrier activity, heme binding
1017	Thiol peroxidase	–	Cytoplasmic	Cellular response to oxidative stress	Thioredoxin peroxidase activity
1023	Hypothetical protein RBAM_028130	–	Cytoplasmic	Unknown	Unknown
1092	Transcriptional repressor CodY	–	Cytoplasmic	Transcription, DNA-dependent	DNA binding, GTP binding, sequence-specific DNA binding, transcription factor activity
1250	Hypothetical protein Dtox_1245	–	Cytoplasmic	Unknown	Unknown
1295	Galactose-1-phosphate uridylyltransferase	–	Cytoplasmic	Galactose Metabolic Process	UDP-glucose: hexose-1-phosphate uridylyltransferase activity
1366	M6 family metalloprotease	–	Extracellular	Proteolysis	Metallopeptidase activity

^a^ Spot numbers assigned by the software refer to the proteins labeled in Figure 1; ^b^ Protein name in the National Center for Biotechnology Information (NCBI) database for *Bacillus amyloliquefaciens*; ^c^ Cellular localization of proteins; ^d^ Clusters of orthologous groups; ^e^ Biological process was assigned according to the protein knowledge base (http://www.uniprot.org) [16] for *Bacillus amyloliquefaciens*; ^f^ Molecular functional annotation was assigned according to the protein knowledge base (http://www.uniprot.org) [16] for *Bacillus amyloliquefaciens*; and ^g^ not in clusters of orthologous groups (COG).

### 2.5. Gene Expression Verification by qRT-PCR

**Figure 3:**
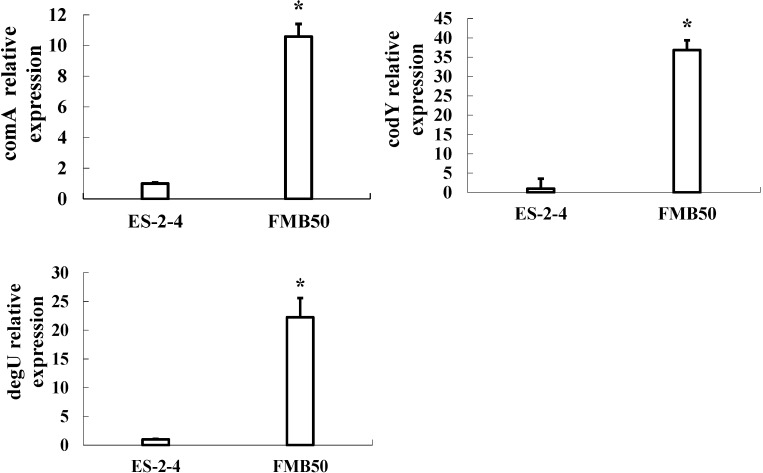
qRT-PCR analysis of mRNA expression of *comA*, *degU* and *codY* genes. Asterisks indicate a statistically significant difference (*p* < 0.05) between the parental strain ES-2-4 and recombination strain FMB38.

Expression of the three genes encoding differentially expressed proteins related to surfactin synthesis (*comA*, *codY*, and *degU*) was analyzed by qRT-PCR analysis of mRNA from FMB38. The mRNA expression profiles of these genes are shown in Figure 3. The mRNA levels of *comA*, *codY* and *degU* were upregulated in FMB38. The upregulated expression of *comA* and *codY* mRNA in FMB38 agreed with their protein levels, although *degU* showed upregulation at the transcriptional level, but was downregulated at the protein level. This disparity was probably due to a methodological error, different regulation of the rates of mRNA and protein turnover, or coverage of several protein isoforms or several members of a gene family [16].

## 3. Discussion

### 3.1. Proteins Related to Surfactin Synthesis

**Figure 4:**
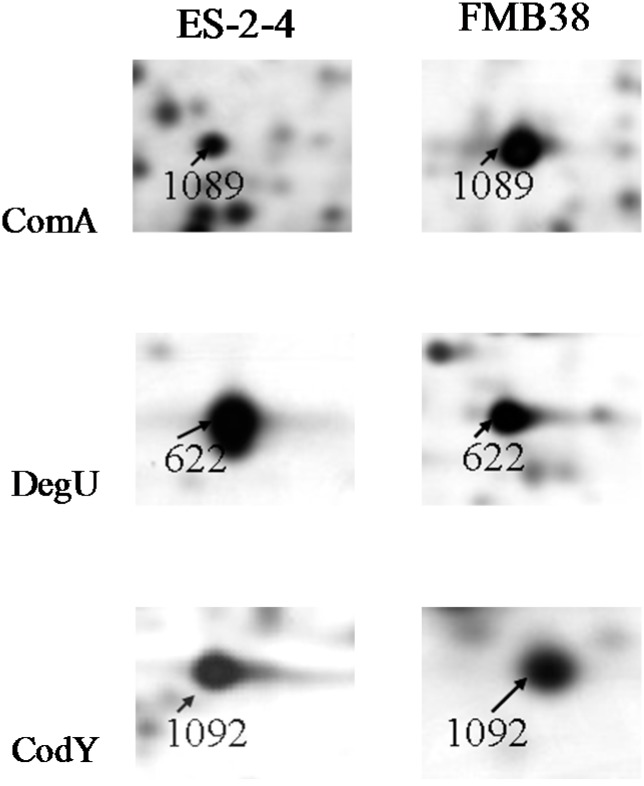
Enlargement of ComA, DegU, and CodY protein spots on 2-DE gels.

**Figure 5:**
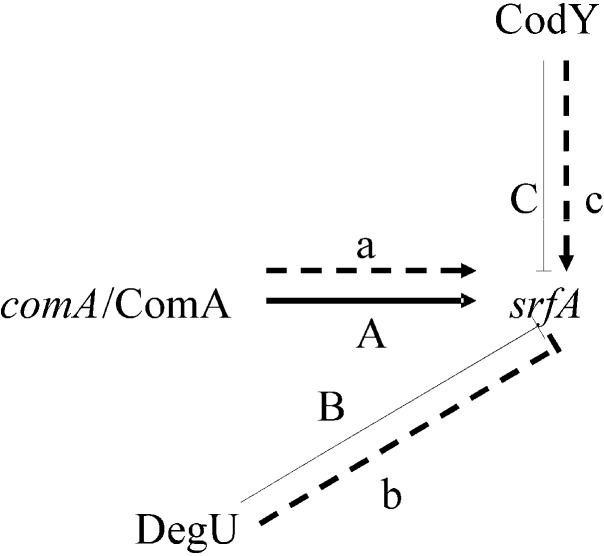
Simplified scheme showing some of the regulators of surfactin synthesis and the roles of ComA, DegU, and CodY in positive (→) and negative (┤) regulation. The solid lines and capital letters show the regulation has been confirmed in the literature, the dash lines and small letters show the regulation speculated in this study.

Of the 46 identified differentially expressed proteins, ComA, DegU, and CodY were directly related to surfactin synthesis. ComA and CodY were upregulated in FMB38, while DegU was downregulated. The three spots corresponding to these proteins on the 2-DE gels are shown in Figure 4. Marahiel *et al.* [17] reported that ComA acts as a transcriptional regulatory protein, and can directly bind to the *srfA-D* promoter, thus promoting the synthesis and secretion of surfactin (Figure 5A). DegU/DegS is an important regulatory protein, and plays a role in many physiological activities of the cell, including the secretion of extracellular proteases, cell migration, and competence. As a protein kinase regulatory protein, both phosphorylated and unphosphorylated DegU are also involved in the physiological activity of the cell. Phosphorylated DegU stimulates the synthesis of extracellular protease and inhibits the expression of *sigD*. However, in its unphosphorylated state, DegU can stimulate *comK* expression, thereby triggering cell competence. In *B. subtilis*, DegU/DegS and ComA/ComP act as a molecular switch that controls the occurrence of cellular changes in different physiological states. The physiological state in the later phases of cell growth is closely linked to the synthesis and secretion of antimicrobial substances. Hahn *et al.* [18] reported that DegU inhibited *srfA* operon expression (Figure 5B); However, the specific negative regulatory mechanism remains unclear. Hamoen *et al.* [13], Stein [12], and Serror *et al.* [19] showed that CodY acted as a transcriptional inhibitor of *srfA* expression (Figure 5C). Duitman *et al.* [20] constructed a *codY*-knockout vector to transform *B. subtilis* ATCC 6633. The resulting strain, BV12I38, showed no effect of CodY on surfactin synthesis. In the present study, upregulated ComA and downregulated DegU were consistent with a surfactin production increase, which was stimulated by ComA (Figure 5A), but inhibited by DegU (Figure 5B). The upregulated CodY in FMB38 contradicted the surfactin production increase (Figure 5C), contrary to previous reports. This was possibly because the entire network of metabolic regulation relies on signal factors, which both promote each other and are mutually antagonistic. Therefore, the CodY regulatory mechanism in surfactin synthesis in *B. amyloliquefaciens* requires further study.

### 3.2. Metabolism-Related Proteins

We identified 12 differentially expressed proteins related to the metabolism of carbohydrates, lipids, amino acids, nucleic acids, and coenzymes. A further two proteins were involved in inorganic ion transport and metabolism, and one protein was associated with co-metabolism, biosynthesis, transport, and catabolism.

The synthesis levels of key enzymes involved in glycolysis and the pentose phosphate pathway of glucose metabolism were higher in the genome-shuffled strain. The putative implications of these increases are described below. Fructose-bisphosphate aldolase is an important enzyme in the glycolysis pathway of all organisms. It catalyzes the cleavage of 1,6-diphosphate-d-fructose to 3-phospho-d-glyceraldehyde and α-dihydroxyacetone phosphate, and can also catalyze the reverse reaction in gluconeogenesis. Glyceraldehyde-3-phosphate dehydrogenase catalyzes glyceraldehyde-3-phosphate dehydrogenation and phosphorylation to generate 1, 3-diphosphoglycerate with a high-energy phosphate bond. The released hydrogen and electron are then transferred to NAD^+^ by the dehydrogenase coenzyme, to form NADH. The phosphate radical comes from inorganic phosphate. Phosphopyruvate hydratase catalyzes the reaction with 2-phosphoglycerate to generate phosphoenolpyruvate. Glucose can be degraded in the glycolytic pathway to generate ATP and provide raw materials for the synthesis reaction. Transaldolase in the non-oxidative stage of the pentose phosphate pathway can catalyze the reaction between glyceraldehyde-3-phosphate and 7-sedoheptulose monophosphate to generate 4-phosphate erythrose and fructose 6-phosphate. NADP produced in the pentose phosphate pathway could provide reducing power for the biosynthesis reaction, while pentose phosphate generated in this pathway could then participate in nucleic acid metabolism.

Pyrroline-5-carboxylate reductase is an important enzyme involved in the reduction of pyrroline-5-carboxylate to proline. Because of this function, it was widely believed that pyrroline-5-carboxylate reductase played an important regulatory function in a series of pathological and physiological processes such as cell apoptosis. 2-Aminoethylphosphonate-pyruvate transaminase belongs to the Valley-Grass transaminase family, and catalyzes the synthesis of aspartate, which is one of the amino acids that constitute surfactin. We speculate that the increased surfactin production by strain FMB38 is a result of increases in this enzyme. The acetoin reductase 2,3-butanediol dehydrogenase (YdjL) encoded by *YdjL* plays an important role in amino acid transport and metabolism [21]. Biosynthesis of many amino acids is closely linked to certain metabolic pathways such as glycolysis and the pentose phosphate pathway. Therefore, substances closely related to amino acid biosynthesis could be regarded as starting materials. Under conditions where the expression of enzymes related to amino acid transport and metabolism are increased, surfactin synthesis will also be enhanced in the recombinant strain. Upregulation of NAD synthetase (NadE) improves NAD generation, and thus would greatly improve the metabolic activities in of enzymes using NAD as a coenzyme in the recombination strain. Thus, we speculate that the increase in antibacterial lipopeptide yield accompanies the increased synthesis of key enzymes of the glycolysis and pentose phosphate pathways. Additionally, the abundance of aspartic acid transaminase and other key enzymes in amino acid metabolism likely enhances surfactin production. Furthermore, expression levels of key enzymes in lipid metabolism, coenzyme metabolism, and inorganic ion metabolism processes were also raised.

### 3.3. Proteins Related to Energy Generation and Transformation

Two of the identified proteins were related to energy production and conversion: the hypothetical protein RBAM_007590, and the ATP synthase α subunit (F0F1 ATP synthase subunit α). Both of these proteins were upregulated in the recombination strain. The ATP synthase α subunit is involved in proton transport processes coupled with ATP hydrolysis and with ATP synthesis. The enhancement of synthesis of enzymes associated with energy production and conversion may cause the increase of the antibacterial lipopeptide yield indirectly.

### 3.4. Proteins Related to DNA Replication, Recombination and Repair

The five DNA replication-related proteins, DNA primase, DNA-directed DNA polymerase III α subunit, recombinase protein, multifunctional SOS repair factor and Site-specific recombinase XerD, are upregulated.

DNA primase is a DNA-dependent RNA polymerase, whose function is to synthesis an RNA primer firstly in the process of DNA replication and carry on to extend DNA fragments in this primer [22], different from the RNA polymerase in the DNA transcription. DNA polymerase III is the necessary enzyme for DNA replication in the cells, having polymerase, exonuclease and endonuclease activities. Replication of the DNA molecule is the synthesis process of progeny DNA with the parental DNA molecule as a template. This process is completed with the chromosome replication in cell mitosis and the first meiosis interphase. The transmission of genetic information is accomplished by the replication of the DNA molecule. Through DNA molecules replication, the genetic information is passed from parent to offspring, thereby ensuring the continuity of the genetic information. The abundances increase of DNA replication-related enzymes could ensure the transmission of genetic information more powerful in the recombination strain.

SOS response is the emergency effects in case of cell DNA injury or copy system inhibited. The SOS response is caused by the interaction of RecA protein and LexA repressor. RecA protein not only play an important role in homologous recombination, but it is also the SOS response factor initially launched. It can be showed that, in the high-yielding strain, DNA serious injury caused by UV, NTG, and ion beam mutagenesis will lead to a series of complex inductive effects, called emergency response. Emergency response induces the excision and reorganization repair enzymes, elevate the levels of these enzymes within the cell and further strengthen excision repair and recombination repair capacity. In addition, SOS reaction can induce the development of DNA polymerase’s lack of proofreading function, speed up repair, and avoid death, while at the same time increase the mutation rate.

### 3.5. Proteins Related to Translation and Post-Translational Modifications

There are three proteins related to translation and post-translational modification respectively. The former includes Tu elongation factor Tu, 30S ribosomal protein S1, Methionine aminopeptidase, type I, while the latter includes alkyl hydroperoxide reductase small subunit, groEL gene product and hypothetical protein RBAM_036960. These six proteins are upregulated in the recombination strain.

Translation elongation factor EF-Tu belongs to the protein elongation factor family and is related to protein synthesis. There are three elongation factor EF-Tu, EF-Ts and EF-G in prokaryotes. The complexes are formed by EF-Tu and the second tt-tRNA integrated with A site of ribosomes, then GTP are hydrolyzed to release, regenerate and form complexes by EF-TS for the next cycle. The protein extending factors Tu/Ts/G are necessary for prokaryotic protein synthesis.

Ribosomes are the place of protein biosynthesis. Ribosome size is represented by the sedimentation coefficient *S*: The larger *S* value, the larger the particle and the greater the molecular weight. There are approximately 20,000 ribosomes in a vigorous growth bacterial, wherein the protein accounts for 10% of the total cellular proteins and rRNA account for 80% of the total cellular RNA. In 1968, the self-assembly of *E. coli* small subunit *in vitro* was researched, and it was found that 30S small subunit with natural activity be formed by adding in 16s rRNA and 21 proteins. In prokaryote 70S ribosome, the 30S subunit (small subunit) contains 22 kinds of the ribosomal protein, and the 50S subunit (large subunit) contains 34 kinds of the ribosomal protein, accounting for 35% of the ribosome. Methionine amino peptidase are involved in protein *N*-terminus processing. Protein biosynthesis occupies an important place in cell metabolism. Protein translation process is very complex, involving almost all types of intracellular RNA and dozens of protein factors. Ribosomes are the factories for protein synthesis, methionine amino peptidase and EF-Tu (translation elongation factor) and play an important role in protein processing, and so the upregulation of three proteins in the recombination strain will more effectively ensure the process of protein translation.

GroEL belongs to a highly conserved protein whose function is to assist the biological macromolecules correct folding, assembly, degradation and transport as well as improving cell stressor tolerance. When bacteria encounter environmental stress (such as heat shock), they will be high expressed within the cytoplasm to enhance bacterial tolerance, and therefore these proteins are also known as heat shock proteins [22]. GroEL belongs to the chaperonin 60 (Hsp60) protein family which includes cpn60, *E. coli* GroES/GroEL and *Helicobacter pylori* GroES/GroEL, *etc.* These proteins are highly conserved, but also produce cross-reactivity. Alkyl peroxide reductase is a key H_2_O_2_-degrading enzyme [23]. It is widely known that most of the protein peptide chain showed the expected biological activity only after certain processing to cause the maturation of the protein. Upregulation of proteins related to post-translational modification can accelerate the maturation process of the proteins and make them bioactive.

### 3.6. Proteins Related to Cell Secretion and Signal Transduction Mechanisms

There are three proteins involved in cell secretion and signal transduction mechanisms. The prokaryotes are mainly regulated at the transcriptional level. The activator protein bind the sequences close to the promoter, the affinity enhancement of RNA polymerase with the promoter, and RNA polymerase activity augmentation. The repressor protein can hinder gene transcription by binding manipulate sequence. As previously mentioned DegU (downregulated) and ComA (upregulated) as a transcription factor, the expression change and surfactin production increase in the high-yield strain is closely related. *S*-ribosylhomocysteinase upregulated in the recombination strain has lyase activity, which is both combined with the iron ions and related to quorum sensing [24]. In addition, the upregulation of ClpP [25] (ATP-dependent Clp protease proteolytic subunit) can improve the cell motility and secretion in FMB 38.

### 3.7. Hypothetical and Unknown Proteins

Fourteen spots subjected to mass spectrometry are identified as hypothetical or unknown proteins. The proteomics research of *B. amyloliquefaciens* is rarely reported and its protein database imperfect. Therefore, further research is needed to obtain the related protein function message in surfactin synthesis process. For the protein function indirectly noted in *B. amyloliquefaciens*, we can refer to its function in *B. subtillis* or other microorganisms to speculate.

All these indicated that the metabolic capability of the mutant was improved by the genome shuffling. The study will enable future detailed investigation of gene expression and function linked with surfactin synthesis. The results of proteome analysis may provide information for metabolic engineering of *B. amyloliquefaciens* for overproduction of surfactin.

## 4. Experimental Section

### 4.1. Strains and Culture Conditions

*B. amyloliquefaciens* ES-2 is an endophytic bacterium isolated from the Chinese medicinal plant *Scutellaria baicalensis* Georgi [26]. *B. amyloliquefaciens* ES-2-4 was obtained by 20 keV N^+^ ion beam implantation [27]. *B. amyloliquefaciens*FMB38 was the genome-shuffled mutant strain of *B. amyloliquefaciens* ES-2-4 [15]. These strains are available from the Key Laboratory of Food Processing and Quality Control of the Food Science and Technology College at Nanjing Agricultural University, Nanjing, China. *B. amyloliquefaciens* ES-2-4 was cultured in standard potato dextrose agar (PDA) media at 37 °C. All microbial strains were maintained in BPY supplemented with 20% (*v*/*v*) glycerol and stored at −70 °C. Seed medium (BPY) (beef extract 5.0 g/L, peptone 10.0 g/L, yeast extract paste 5.0 g/L, glucose 10.0 g/L, NaCl 5.0 g/L) and fermentation medium (glucose 42.0 g/L, l-sodium glutamate 4.0 g/L, MgSO_4_ 0.5 g/L, KCl 0.5 g/L, KH_2_PO_4_ 1.0 g/L, FeSO_4_ 0.15 mg/L, MnSO_4_ 5.0 mg/L, CuSO_4_ 0.16 mg/L) were adjusted to pH 7.0.

### 4.2. Protein Sample Preparation

Cells were statically cultured at 32 °C for 36 h. After harvesting by centrifugation at 6000× *g* for 5 min at 4 °C, pelleted cells were washed three times with 20 mM Tris-HCl (pH 6.8). Cells were subsequently resuspended in lysis solution containing 7 M urea, 2 M thiourea, 4% (*w*/*v*) CHAPS (3-[(3-Cholamidopropyl) dimethylammonio] propanesulfonate), 40 mM DTT (Dithiothreitol), and 2% pH 3–10 ampholytes. Samples were disrupted by sonication in an ultrasonic cell pulverizer (Xin-zhi Biotechnology Co., Ningbo, China), equipped with a cup horn, for 45 min on ice. Following ultra-sonication, Nuclease Mix (GE Healthcare, Little Chalfont, UK) was added to a final concentration of 1% (*v*/*v*). The mixture was incubated for 1 h at room temperature and then centrifuged for 30 min at 13,000× *g* at 4 °C. Protein concentrations of the resulting supernatants were determined using a 2-D Quant kit (GE Healthcare), with bovine serum albumin as a standard. The proteins were stored at −70 °C until required for 2-DE.

### 4.3. 2-DE and Staining

For in-gel rehydration of each sample, 300 µg of protein was dissolved in 130 µL of rehydration buffer containing 7 M urea, 2 M thiourea, 2% (*w*/*v*) CHAPS, 18 mM DTT, 2% Bio-Lyte (Bio Rad, Hercules, CA, USA), and 0.002% (*w*/*v*) bromophenol blue. IEF was performed on an Ettan IPGphor 3 IEF system (GE Healthcare) with 24 cm linear immobilized pH gradient (IPG) strips (pH 4–7, GE Healthcare). The loaded IPG strips were focused at 20 °C and 50 V for 10 h, 250 V for 3 h, 500 V for 3 h, 1000 V for 1 h, and 8000 V for 1 h, followed by 8000 V until a total of 80 kVh was reached. Following separation in the first dimension, the strips were equilibrated in a solution containing 6 M urea, 75 mM Tris-HCl (pH 8.8), 30% (*w*/*v*) glycerol, 2% (*w*/*v*) SDS, and 2% (*w*/*v*) DTT for 15 min at room temperature. The IPG strips were then equilibrated with the rehydration buffer described above, in which the DTT was replaced with 2.5% (*w*/*v*) iodoacetamide, for 15 min at room temperature. The strips were then transferred to 12.5% (*w*/*v*) SDS-polyacrylamide gels. Second dimension electrophoresis was carried out in an Ettan DALTII system (GE Healthcare) with a constant power of 5 W per gel for the first 30 min, followed by 12 W per gel for 6–7 h until the bromophenol blue dye front reached the bottom of the gels. Gels were fixed in a 40% (*v*/*v*) methanol and 10% (*v*/*v*) acetic acid solution overnight, and then stained with 0.25% (*w*/*v*) silver nitrate. At least three biological replicates were performed for each treatment.

### 4.4. Image Acquisition and Data Analysis

The silver-stained 2-DE gels were scanned at 400 dots per inch using an ImageScanner (GE Healthcare), and resulting images were analyzed using ImageMaster 2D Elite software (version 2.00; GE Healthcare) for spot detection, quantification, and comparative and statistical analyses. Images were cropped and optimized, and then gel-to-gel matching of the standard protein maps was performed. The spot detection parameters were optimized by checking different protein spots in certain regions of the gel, then automatically detected as described above, followed by visual inspection for removal or addition of undetected spots. Spot detection was refined by manual spot curation when needed. Spots that were present on at least two gels of one treatment or control were identified as expressed protein spots. The abundance of each protein spot was estimated by normalizing the spot volumes as a percentage of the total volume of all the spots in the gel to correct for any variability due to loading, gel staining, and destaining. Triplicate gels were used for each sample. The volume of each spot on the three replicate gels was normalized to the total spot volume from the reference gel, quantified, and then subjected to one-way ANOVA. Only those spots showing reproducible and significant changes were considered to be differentially expressed proteins.

### 4.5. Protein In-Gel Digestion

Detected spots and four control spots in unstained gel areas were excised (approximately 1 mm^3^ cubes) from silver nitrate-stained gels. For in-gel protein digestion, the gel-bound proteins were washed at room temperature with a 1:1 solution of 50 mM ACN (acetonitrile): NH_4_HCO_3_, once for 10 min and once for 30 min. Gels were dehydrated in 20 μL ACN for 20 min, and then dried in a vacuum centrifuge (Eppendorf, Hamburg, Germany) for 30 min at 30 °C. Proteins were reduced by incubation in 50 μL of 10 mM DTT/25 mM NH_4_HCO_3_ at 56 °C for 1 h, and were then alkylated in 50 μL of 55 mM iodoacetamide/25 mM NH_4_HCO_3_ for 45 min at room temperature in darkness. The liquid was discarded and gel pieces were washed twice in 25 mM NH_4_HCO_3_, dehydrated in ACN, and dried in a vacuum centrifuge for 30 min at 30 °C. Gel pieces were then rehydrated in 4 μL of 25 mM NH_4_HCO_3_ containing 40 ng trypsin, and incubated at 4 °C for 1 h. Excess liquid was discarded and gel plugs were incubated at 37 °C overnight, with tubes inverted to keep gel pieces wet for sufficient enzymatic cleavage. Then, 8 μL of 5% (*v*/*v*) TFA (trifluoroacetic acid) was added and samples were incubated at 37 °C for 1 h. Supernatants were collected and the proteins were extracted twice by incubating the gel pieces in 8 μL of 2.5% TFA/50% ACN at 37 °C for 1 h. Supernatants were mixed and completely dried in a vacuum centrifuge. Resulting peptides were stored at 4 °C until further use.

### 4.6. Protein Identification by MALDI-TOF and Database Searches

Dried peptides were dissolved in 2 μL of 0.5% TFA. The matrix material was dissolved until saturated in TA solution (ACN:0.1% TFA:acetone = 3:6:1). The matrix and the analyte solution were mixed at a ratio of 1:1, then 1 μL of the mixture was deposited onto the stainless steel sample target, and the solvent was allowed to evaporate at ambient temperature. MALDI-TOF analyses of trypsin digests were performed on a Biflex IV MALDI-TOF-MS (Bruker, Billerica, MA, USA) equipped with a N_2_ laser (337 nm, 3 ns pulse length) in positive ion mode at an accelerating voltage of 19 kV. Peptide data were collected in reflectron mode. Each spectrum was the accumulation of approximately 200 laser shots. External calibration was performed using peptide calibration standards.

Data were compared with the National Center for Biotechnology Information (NCBI) nr database using the MASCOT search program (Matrix Science, Boston, MA, USA). The following parameters were allowed: taxonomy restrictions to other firmicutes, one missed cleavage, 120 ppm mass tolerance in MS, carbamidomethyl (C) as a fixed modification, and oxidation (M) as a variable modification. The confidence in the peptide mass fingerprinting matches (*p* < 0.05) was based on the MOWSE score and confirmed by accurate overlap of the matched peptides with the major peaks of the mass spectrum. Only significant hits, as defined by the MASCOT probability analysis (*p* < 0.05), were accepted.

### 4.7. RT-PCR Analysis

Total RNA was extracted from *B. amyloliquefaciens* cultures using TRIzol Reagent (Invitrogen, Carlsbad, CA, USA) according to the manufacturer’s instructions, and then treated with RNase-free DNase (Biomiga, Santiago, PA, USA). First-strand cDNA was synthesized from total RNA using an RT-PCR kit (Fermentas, Vilnius, Lithuania). Real-time PCR was performed using a real-time PCR SYBR Green Master Mix kit (Toyobo Biologics, Osaka, Japan) on a Rotor-Gene 3000 RT-PCR System (Corbett Research, Sydney, Australia). Each 25 µL PCR reaction contained 10 µL of real-time PCR mix, 1 µL of template DNA (100 ng), and 2 µL of 10 µM primers (Table 3). The PCR conditions were as follows: 95 °C for 5 min, followed by 40 cycles of 95 °C for 15 s, 60 °C for 30 s, and 72 °C for 30 s. Each reaction was performed in triplicate. Following threshold-dependent cycling, melting was performed from 45–95 °C at a 0.1 °C/s melt rate with a smooth curve setting averaging one point. Primer specificity was verified by melt curve analysis. Band intensities were normalized to the 16S rDNA transcript band for 2^−ΔΔ*C*t^ relative quantification. The *B. amyloliquefaciens* nucleotide sequences for these genes were obtained from the NCBI GenBank database. Primer pairs were designed from these sequences with Primer Premier 5.0 software (Premier Biosoft, Palo Alto, CA, USA; Table 3).

**Table 3:** Primers used for qRT-PCR.

Gene	Forward Primer (5'→3')	Reverse Primer (5'→3')
16S rDNA	CCTACGGGAGGCAGCAG	ATTACCGCGGCTGCTGG
*comA*	TCAAAGTGAGCAGGATCGGTTAA	CTTCTGTACGGGAGCCGACAT
*codY*	GGCAGGCAAACCCGTAAACT	ACTGGCGGTCTTCCAGCATT
*degU*	CACCCGAAAGTAACCCACAAT	AGCACTTCACATTCCCGTCTC

## 5. Conclusions

In conclusion, the functions of the identified differential proteins appeared to be interconnected. The results revealed that the improvement in lipopeptide yield in the genome-shuffled strain was not the result of a single metabolic pathway or a small number of proteins, but a combination of gene transcription and translation processes, sugar, protein, and energy metabolism, and many other related factors. Of the 46 identified proteins, ComA, DegU, and CodY were directly related to surfactin synthesis, and we determined that ComA and CodY stimulate surfactin synthesis, while DegU acts as an inhibitor. The integration of proteomic information could offer more rational strategies for the genetic modification of *B. amyloliquefaciens* cells with an enhanced surfactin synthesis capacity.
